# Touch Piezoelectric Sensor for Vibration Intensity Testing

**DOI:** 10.3390/s25196196

**Published:** 2025-10-06

**Authors:** Algimantas Rotmanas, Regimantas Bareikis, Irmantas Gedzevičius, Audrius Čereška

**Affiliations:** Department of Mechanical and Material Engineering, Faculty of Mechanics, Vilnius Gediminas Technical University (VILNIUS TECH), Plytinės Str. 25, LT-10105 Vilnius, Lithuania; algimantas.rotmanas@vilniustech.lt (A.R.); regimantas.bareikis@vilniustech.lt (R.B.); irmantas.gedzevicius@vilniustech.lt (I.G.)

**Keywords:** piezo sensor, vibration testing, piezoelectric materials, ultrasonic system

## Abstract

The article presents research on a wide frequency range piezo sensor applied to surfaces by touch. It details the design of the piezo sensor, its operating principles, and usage characteristics. Calculations of the main vibration forms and modes, modeling, and experimental verifications are provided. The objective of the research was to create a lightweight, ergonomic device that enables quick detection and testing of ultrasonic vibrations on objects (ultrasonic concentrators, their replaceable tips, concentrator mounting structures, device casings, etc.) with a brief touch—up to 1 s. After optimizing the design parameters and conducting tests, it was determined that the piezo sensor identifies vibrations in the range of 20–96 kHz, which is a commonly used range in ultrasonic vibration systems (UVS). A distinctive feature of the sensor is that in this frequency range, it does not generate amplitude peaks, and its structural elements do not enter into the resonances of lower modes (1–5). The piezo sensor is not intended to determine precise vibration amplitudes and forms. It is designed to quickly find the points of minimum and maximum vibrations in vibrating objects, where precise measurements will later be conducted. The conducted research will assist in the design and manufacturing of such devices.

## 1. Introduction

In conducting research on objects vibrating at ultrasonic frequencies—such as ultrasonic vibration systems and their components, casings in which they are mounted, and objects affected by ultrasonic concentrators, etc.—a common problem encountered is the lengthy duration of preliminary measurements of vibrating objects. Numerous tests and measurements are required at different device locations before characteristic points can be identified for detailed measurements. For instance, UVS with complex geometric shapes vibrating simultaneously in several vibration forms and higher modes can have many characteristic points—from several to dozens.

Moreover, such testing is highly relevant not only in scientific research but also in industry, when it is necessary to determine the causes of UVS malfunctions or simply carry out routine quality control. This is especially crucial in mass production, where there is no possibility to halt the production process for a longer time during quality control or minor repairs. It is not always feasible to use a laser vibrometer due to the inconvenient location of vibrating parts in the device, non-reflective surfaces, or surfaces covered with a thin layer of liquid, etc. In such cases, it is most practical to use pencil-type sensors [1], which allow for the quick checking of dozens or even hundreds of characteristic points by touch within a short time.

For vibration measurement, existing sensors are being improved, and new ones of various designs and operating principles (capacitive, inductive, optical, inertial, piezoelectric) are being developed [2]. The areas of application are also diverse—including medicine, science, transport, industry, education, etc. However, for several decades, we have observed a trend where such devices (as well as most measuring and other devices) are developed and improved in just a few aspects—increasing sensitivity and precision and aspects of device miniaturization [2,3,4]. Regarding the search for new measurement methods, for example, in works [5,6], vibration sensors using a resonating vibration concentrator have been researched and improved. The property of the concentrator is utilized, whereby merely touching it causes the concentrator to move out of resonance and thus sensitively records changes in electrical signals. In work [7], not only the direct but also the reverse piezoelectric effect is used to amplify the electrical signal, combining it with the direct piezoelectric effect. Much attention is given to the search for new materials and their application in sensors, including piezoelectric ones [8,9,10,11]. Regarding application areas, the majority of attention is given to medical diagnostics [2,8,10], robotics [5,9], and human and device interfaces [10,11,12,13,14].

Unfortunately, the market lacks inexpensive, simple, yet universal tools for vibration testing that could be used for preliminary tests before conducting detailed measurements. In this work, a prototype tool that could be considered is a surface roughness testing tool [15], operating on the principle of the direct piezoelectric effect. However, the difference is that for the prototype [15], entering into resonant modes is not relevant. In our case, the piezo sensor entering into resonance would significantly distort the information, so theoretical modeling and experimental research of the piezo sensor junction were conducted with the aim of minimizing the excitation of intrinsic vibrations in the sensor.

## 2. Design of Piezo Sensor

**Figure 1:**
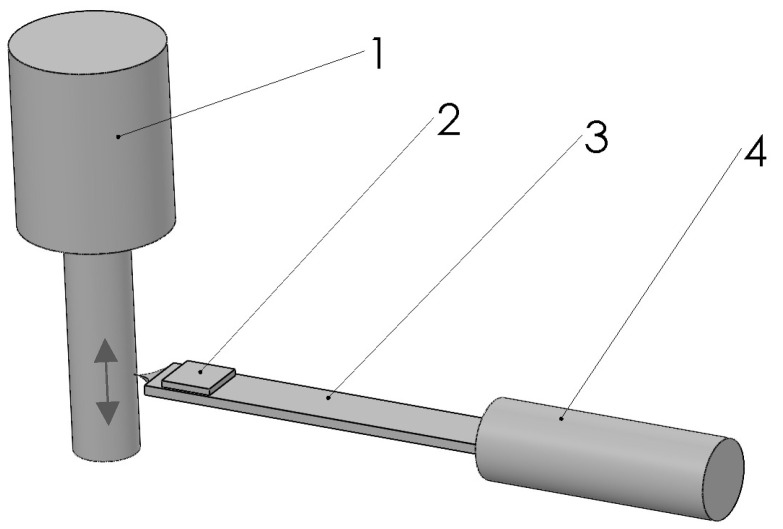
Schematic diagram of the piezo sensor: 1—the object being tested that is vibrating; 2—sensor “head” with a needle and piezoceramic; 3—steel joint (the connection); 4—holder.

When designing the piezo sensor, contact measurement methods were used, which have the advantage of allowing the piezo sensor’s needle to be mechanically connected to the measured object (simply by pressing the needle tip briefly, up to one second, at the required point on the vibrating measured object (Figure 1)). Of course, the needle must be sharp enough, and the material being tested must not be harder than the material of the needle.

Depending on the orientation of the piezo sensor relative to the direction of vibration displacements, an electrical signal is obtained from the output of the piezo element, which is proportional to the component of the vibration displacements in that direction.

During the assessment of various tested vibrating objects for their minimum and maximum amplitudes and frequencies, several “pencil” type touch sensors were designed, modeled, and constructed. The chosen design featured a sensitive element, a piezoceramic plate, mounted in the “head” of the piezo sensor, to which a needle contacting the tested object was attached, while the opposite side of the head was connected to the piezo sensor holder via a connecting link. The connecting link could be either a straight or S-shaped bent steel plate. Since the head is directly mechanically pressed against the vibrating object, to minimize influence, it needs to be as small and light as possible. Therefore, the piezoceramic plate is also minimized to dimensions that do not require additional filtering, amplification, or other processing of the generated signal, as such treatment could distort the characteristic displacement and acceleration points of the tested object. The electrical signal from the piezo plate is directly connected to an oscilloscope or another electrical signal recording device. During the preliminary design and modeling phase, it was determined that the optimal form of the piezoceramic is a square plate 0.2 mm thick, weighing about 8 mg, with the head including the plate and needle weighing about 33 mg.

During the testing of the first prototype, it was discovered that the main issue with such a piezo sensor is the vibration of the connection. As the needle and the head vibrate forcefully together with the tested object, the vibrations also transfer to the connection. The other end of the connection, which is attached to the holder, can theoretically be considered as being fixed in place. Therefore, the head-connection system can be viewed as a vibration system, where the head acts as the vibration initiator. The connection must be flexible; otherwise, it would significantly dampen the vibrations and greatly weaken the electrical signal. A flexible connection is also necessary for reliable pressure application of the needle to the testing point. Structurally, a steel connection is most rational, but a certain form of steel body can enter into resonance and severely distort the tested data. Initially, the material and cross-sectional shape of the connection were selected. Preliminary modeling determined that spring steel is best suited, and considering the direction of the initiating vibrations and the orientation of the piezoceramic plate, a rectangular cross-section is most appropriate.

## 3. Mathematical Modeling of the Sensor’s Connection

**Figure 2:**
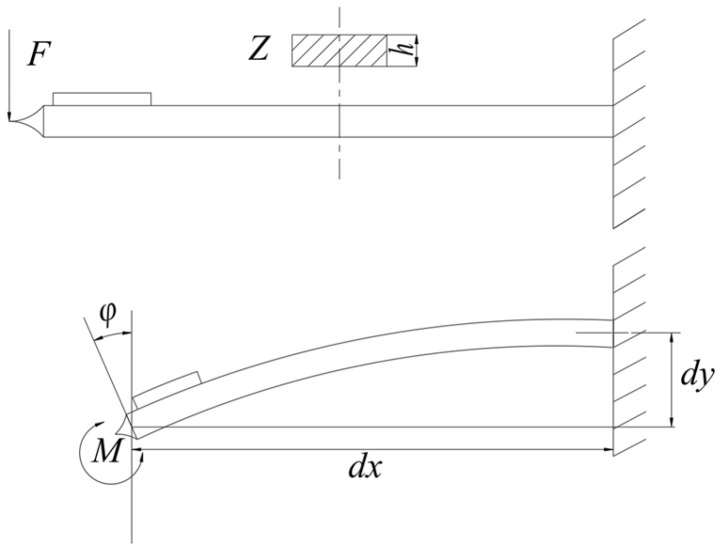
Diagram for calculating the sensor’s connection.

In a rectangular cross-section connection, when vibrations are initiated perpendicular to the longer side of the rectangle, bending vibrations are primarily induced (Figure 2).

When a force *F* acts on the sensor’s needle, bending deformation occurs in the connection (a beam with a rectangular profile) at an angle φ. The moment of force *F*, *M* (Figure 2) [16] can be expressed as follows:

(1) $$M=-EI\frac{dS}{dx}=EI\frac{d^{2}y}{{dx}^{2}}\;,$$

the force *F* can be expressed as follows:

(2) $$F=\frac{dM}{dx}=EI\frac{d^{3}y}{{dx}^{3}}$$

here *E* is Young’s modulus, and *I* is the moment of inertia with respect to the *z*-axis (Figure 2).

To derive the equation of motion, the force *F* needs to be expressed in terms of mass and acceleration, and the mass needs to be expressed in terms of the density *ρ* of the connection material, the cross-sectional area *S*, and the coordinate *x*. By expressing the force *F* this way and substituting it into Formula (2), we obtain the equation of motion:

(3) $$\frac{d^{4}y}{dx^{4}}+\frac{\rho }{EK^{4}}\cdot \frac{d^{2}y}{{dt}^{2}}=0$$

The obtained Equation (3) is the equation of motion for the sensor’s connection in the form of bending vibrations, and such equations are commonly used for calculating harmonic bending elastic deformations [16,17]. The term *K* is the radius of gyration of the sensor connection’s cross-sectional inertia:

(4) $$K=\sqrt{\frac{I_{z}}{S}}$$

The cross-section of the sensor’s connection is rectangular; therefore, the Formula (4) for a rectangle or square shape will be as follows:

(5) $$K=\frac{h}{\sqrt{12}}$$

Based on [15,16], Formula (3) is suitable for calculating joints where the height h is significantly smaller than the length. However, we will also calculate higher modes of bending vibrations, not just the first one. As the frequency increases, the wavelength decreases, so it can decrease to the extent that *h* becomes close to the wavelength. Therefore, we refine Formula (3) based on [16] as follows:

(6) $$\frac{d^{4}y}{dx^{4}}+\frac{\rho }{EK^{4}}\cdot \frac{d^{2}y}{{dt}^{2}}=-\frac{\rho }{E}\cdot \frac{d^{4}y}{{dx^{2}dt}^{2}}=0$$

The connection will experience harmonic vibrations (or very close to harmonic, whose motion can be considered sinusoidal), therefore the solution to Equation (3) [16] is as follows:

(7) y=Achnx+Bchnx+Ccosnx+Dsinnx

where $n=\sqrt{\frac{\omega }{vK}}$;

*ω* is angular frequency, and v is the speed of sound.

Solution to Equation (6):

(8) y=Acos∝x+Bsin∝x+Cchβx+Dshβx

where *A*, *B*, *C*, *D* are coefficients dependent on the boundary conditions of the vibrating connection’s ends.

Since in the case of the piezo sensor connection vibrations, the value of *K*^2^ is relatively very small compared to $\frac{\omega^{2}}{2v^{2}}$, we assume the condition that ∝=β=n; $n=\sqrt{\frac{\omega }{vK}}$.

As highlighted, the operation of the piezo sensor would be most disrupted by resonant vibrations of the connection, excited by the vibrations of the objects being tested. Therefore, it is practical to calculate the resonance frequencies for several main conditions:

When the end of the connection is supported:

$$y=0;\;\frac{d^{2}y}{d^{2}x}=0;\;x=0\;\mathrm{or}\;x=l.$$

When the end of the connection is fixed (assuming that the end of the connection in the holder is immovably fixed and the mass of the holder is significantly greater than the mass of the connection):

$$y=0;\;\frac{dy}{dx}=0;\;x=0\;\mathrm{or}\;x=l.$$

When the end of the connection is free (in cases where the piezo sensor’s head vibrates in phase with the connection):

$$\frac{d^{2}y}{dx^{2}}=0;\;\frac{d^{3}y}{dx^{3}}=0;\;x=0\;\mathrm{or}\;x=l.$$

It is impossible to precisely predict the conditions that may arise at the ends of the connection when the piezo sensor’s needle is supported against the tested object, therefore the vibration Equation (8) should be solved for several conditions:

The solution when both ends of the connection are supported [16]:

(9) $$f_{k}=\frac{\pi }{2}\cdot \frac{k^{2}}{l}vK$$

where *k* is the mode order number 1, 2, and 3.

The solution when both ends of the connection are fixed:

(10) $$f_{k}=\frac{m_{k}^{2}vK}{2\pi l^{2}}$$

The solution when both ends of the connection are free (an unlikely condition, but necessary for a complete picture of the process, so we will calculate the fundamental frequency):

(11) $$f_{1}=3.59\frac{vK}{l^{2}}$$

The solution when one end is supported and the other end is fixed. Theoretically, these are the most likely working conditions for the connection:

(12) thnl−tgnl=0

The first two solutions are $m_{1}=3.93$; $m_{2}=7.07$.

When *k* > 2

(13) $$m_{k}=\frac{4k+1}{4}\pi$$

**Figure 3:**
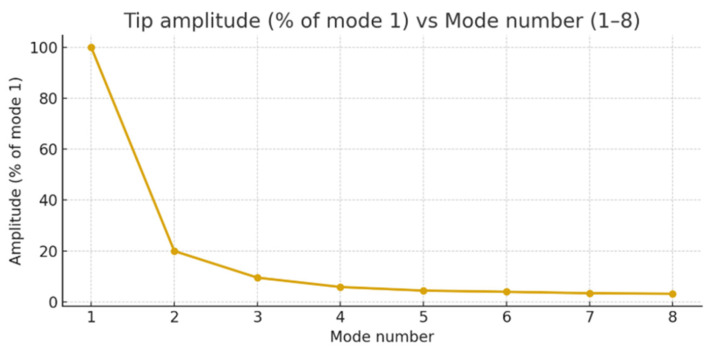
Relative dependence of the oscillation amplitudes of the first mode and higher modes, comparison of the amplitudes of the first mode (100%) and higher modes (up to 8 modes).

The resonant frequency of higher modes is calculated according to Formula (10) by inputting the values of *m_k_* from (13). In oscillatory systems, the amplitude decreases exponentially with increasing mode (Figure 3), so the amplitudes of oscillations above the fifth mode will be approximately 20 or more times smaller than the amplitude of the first mode. This means that mode 6 and above cannot affect the readings of the sensor being tested (including attenuation, frequency shift, energy dissipation, etc.) with an error of more than 3%.

Table 1 presents the calculation results for a steel junction (steel DIN C60) with a length of 30 mm and a cross-sectional height of 0.2 mm. It can be observed that the most “dangerous” and potentially influential modes for the piezoelectric sensor are those within frequency ranges that are significantly distant from the operating frequency ranges of the ultrasonic testing (UT) procedure.

**Figure 4:**
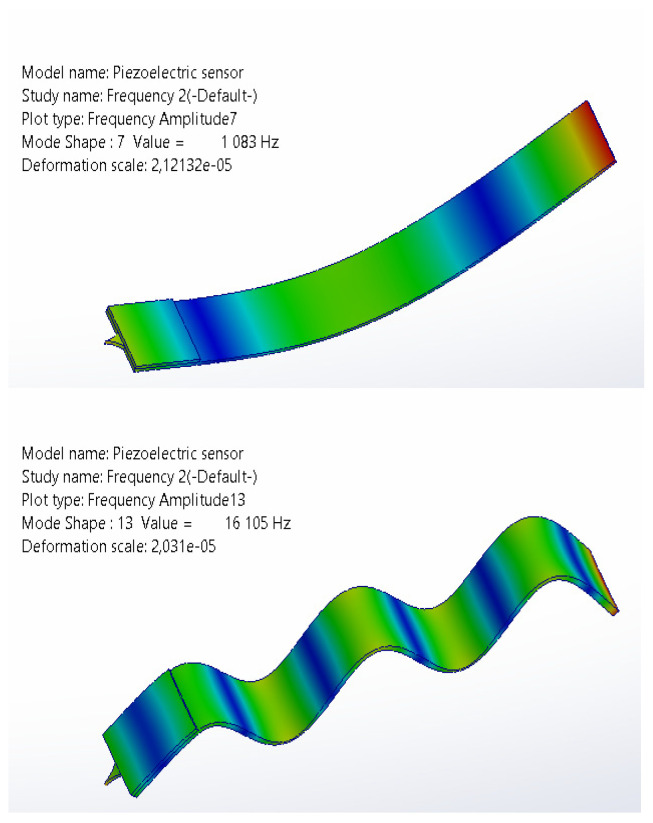
Results of the modeling of first and fifth mode bending vibrations.

The most relevant scenario for sensor operation is when one end of the junction is fixed in the sensor holder, while the other end is supported against the test specimen. Even the fifth mode frequency is considerably lower than the minimum test frequency. Theoretically, this means that the vibrations of the test object should not cause resonant oscillations in the connection, the oscillation amplitudes of which could significantly affect the testing process. These calculation results were compared with the results of numerical experiments, where the piezoceramic and needle were incorporated into the junction structure (see Figure 4); hence, the natural frequencies were slightly lower than the calculated ones. For example, for the first mode, when the junction ends are free, the resonant frequency is 1194.8 Hz. In the modeled junction with piezoceramics and a needle, the resonant frequency of the first mode is 1083 Hz. It is understandable that with additional weight on the vibrating strip end, the frequency is lower. In the case of the sensor, this is an advantage. However, for higher modes, such as modes 2–5, the frequencies are higher than those calculated for junctions without piezoceramics and needles. The frequency of the fifth mode is correspondingly 15,805 Hz (calculated for the junction only) and 16,105 Hz (modeled for the junction with piezoceramics and needle). This can be explained by the fact that in the vibrating junction, the piezoceramics act as additional mass in the first mode, while in higher modes, it increases stiffness and effectively shortens the total length by about 4 mm, vibrating according to the calculated parameters. Incidentally, after modeling the junction without the needle and ceramics, the results of calculations and modeling were consistent within a margin of negligible error.

## 4. Experimental Research

Theoretical calculations and junction modeling results indicate that after preliminary modeling, selected parameters (transducer mass, junction geometry and material, junction fixation conditions, etc.) are suitable for such a sensor. However, everyone working with ultrasonic systems knows from experience that even in simple construction systems, unforeseen vibration shapes and modes sometimes occur. It is also difficult to predict in advance the impact of the transducer head construction on sensor operation. Therefore, an experimental study was conducted.

For the experimental research, a piezoelectric sensor was constructed, with the junction made of steel DIN C60. The junction was mechanically cut from the steel without using lasers or other technologies that could affect the metal’s structure and properties. A piezoceramic plate measuring 4 × 4 × 0.2 mm was cut from PIC 181 piezoceramic. The plate was bonded to the transducer head using composite adhesives. A 0.03 mm^2^ cross-sectional varnished multicore insulated wire was soldered to the piezoceramic plate for electrical signal detection. Signal generation was performed using a Tektronix CFG280 generator; the formation of self-vibrations was observed and recorded using an impedance analyzer HP 4192A; oscillograms were observed using a Matrix MOS-620 oscilloscope. Vibration amplitudes were measured using a “Polytec” laser vibrometer with an OFV-5000 scanning sensor head.

Three methods were tested to introduce vibrations into the sensor connector: (1) The reverse process of the sensor performing direct functions. When the sensor performs a direct testing function, electrical vibrations are generated by mechanical vibrations. During the study, an electrical signal was introduced into the piezoceramic plate, and it generated mechanical vibrations in the sensor connector; (2) The mechanical vibrations occurred by simulating the sensor’s operation directly performing its intended function—testing objects vibrating at a known frequency. Several ultrasonic systems operating in the 20–100 kHz range were selected and tested. The points vibrating with minimum and maximum amplitudes were determined; (3) The points were determined by simulating the real sensor operation and simultaneously measuring the vibration amplitudes on real ultrasonic concentrators with a laser vibrometer in order to determine the correlation between the laser vibrometer readings and the sensor’s electrical signal.

**Figure 5:**
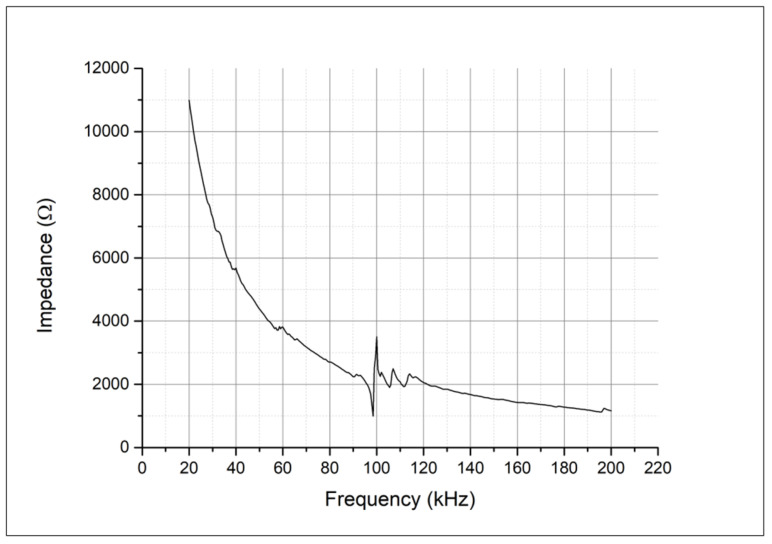
Impedance frequency characteristics of the piezoelectric sensor from 20 kHz to 200 kHz.

We will discuss the results in the same order as presented above, according to the nature of vibration input: scanning was performed with an impedance analyzer, and frequency varied from 20 kHz to 200 kHz. The formation of resonant vibrations was deduced from impedance frequency characteristics. In Figure 5, the curve is shown when scanning in the ultrasonic frequency range from 20 kHz to 200 kHz. The resonance of the junction is clearly visible at 100 kHz. In the range of 20 kHz to 96 kHz, there are no significant changes that could affect the sensor’s operation. Incidentally, it is characteristic that from approximately 120 kHz to 200 kHz, there are also no significant resonances in the piezoelectric sensor junction, so it is likely that the sensor could perform its functions in this range as well.

**Figure 6:**
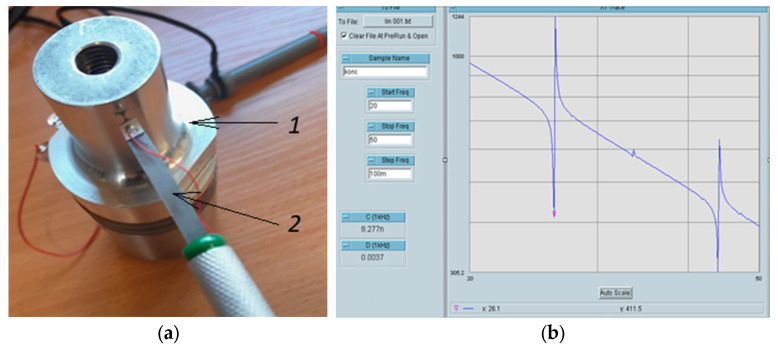
Tested UT system. (**a**) Overall view of the UT system: 1—UT system; 2—piezo sensor; (**b**) Impedance frequency characteristics of the UT system.

**Figure 7:**
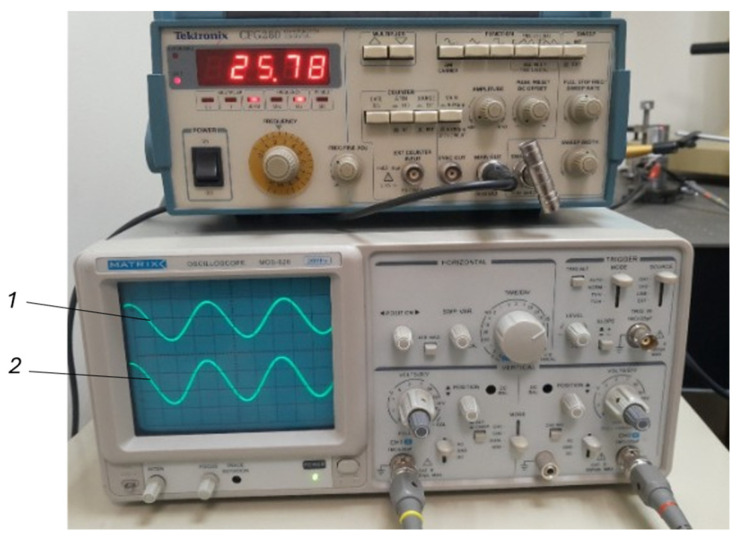
Results of the UT: 1—Signal generated by the piezoelectric sensor; 2—Power signal of the UT system. An example is provided when the system under test vibrates at the resonant frequency (25.78 kHz); the shape and frequency of the piezo sensor signal correspond to the shape of the system power signal (system power supply voltage 40 V; piezo sensor output signal 42 mV).

In simulating the sensor’s operation under real conditions, several UT systems operating at different frequencies (with various shapes and modes) ranging from 20 kHz to 100 kHz were selected. In Figure 6, one of the tested systems operating at 26 kHz (first mode) and 44 kHz (second mode) frequencies is shown. Figure 7 illustrates how the operating frequencies and vibration phases of these systems align with those of the piezoelectric sensor. Systems operating at approximately 20 kHz, 26 kHz, 34 kHz, 44 kHz, 70 kHz, and 96 kHz frequencies were tested. At all these frequencies, the readings of the piezoelectric sensor correlated very well with the vibration frequencies, phases, and amplitudes of the tested systems.

**Figure 8:**
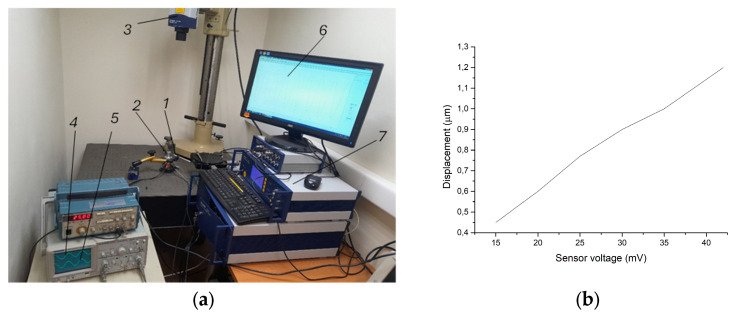
(**a**) The stand, which was used to observe how the real amplitudes of the ultrasonic vibration system and the electrical output signal of the piezo sensor correlate with each other. 1—piezo sensor; 2—the vibrating object under test; 3—“Polytec” scanning sensor head OFV-5000; 4—oscillogram of the electrical signal activating the vibrating object; 5—oscillogram of the electrical signal of the piezo sensor; 6—the amplitude of the displacements of the vibrating object is recorded by the laser vibrometer (shown separately in Figure 9); 7—“Polytec” laser vibrometer. (**b**) Dependence of the electrical signal of the piezo sensor on the amplitude of the vibrations.

In order to determine the correlation between the laser vibrometer readings and the sensor’s electrical signal, a scheme was assembled (Figure 8a), in which the sensor needle is supported on characteristic points of vibrating objects and the electrical signal of the piezo sensor is recorded by an oscillograph. At the same time, the real amplitude of vibrations at these points is measured by the laser vibrometer. Various ultrasonic systems vibrating in both resonant and non-resonant modes were used for the experiments. The way in which these dimensions (laser vibrometer readings and electrical signal voltage) correlate with each other was observed. For example, when testing a system vibrating at a frequency of about 26 kHz, an almost linear dependence of the electrical signal on the amplitude was obtained in the amplitude range from 0.4 to 1.2 μm (Figure 8b).

As the amplitude increased to 8 µm, the relationship was no longer linear, but a clear correlation between amplitude and voltage remained. When testing systems operating in the range up to 96 kHz, a clear correlation between amplitude and voltage was also observed.

**Figure 9:**
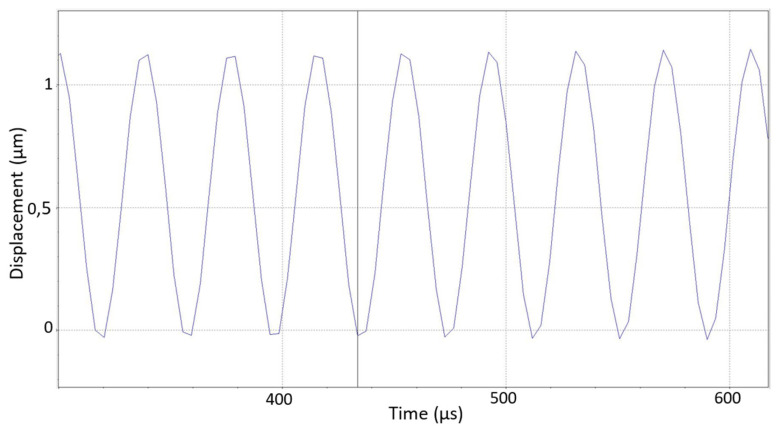
Diagram of displacements of the measured point of the ultrasonic vibration system, recorded by a laser vibrometer. The amplitude of the vibrations is about 1.2 μm.

The effect of pressing force on the operation of the piezoelectric transducer was also studied. It was preliminarily established that a pressing force of 0.8–1.2 N is optimal. However, in some cases, when testing ultrasonic systems weighing more than 150 g, pressing forces of up to 1.8 N had minimal impact on the test object. When testing objects weighing less than 55 g, the influence of the piezoelectric transducer pressing force was too significant, leading to partial or complete de-resonance of the system (if the system under test was operating in resonant mode) or to a significant slowdown of oscillations in the system under test (if the system was not resonant). It is clear that the frequency of vibrating systems is also slightly adjusted due to the influence of the piezoelectric transducer’s touch (even when the tested systems weigh more than 55 g).

However, as mentioned previously, the piezoelectric transducer is not designed for precise amplitude measurements. It is intended to quickly identify areas of higher and lower vibration intensity by comparing short touches, where detailed and precise measurements can then be taken using other instruments. Incidentally, it was previously mentioned that the piezoelectric transducer could theoretically function in the range of 120 kHz to 200 kHz, but this hypothesis has not been confirmed. This is because, when objects vibrate at frequencies above 100 kHz, a reliable mechanical contact between the stylus and the test object is not established.

## 5. Conclusions

Presented is a “pencil” type piezoelectric sensor design, consisting of only three main structural components. The sensor is designed for quick and easy (up to 1 s duration) testing of various ultrasonic vibrating systems, seeking characteristic points vibrating with minimal and maximal amplitudes. Such a sensor helps save time when it is necessary to preliminarily identify points for further detailed measurements using other means.

Calculations of bending vibrations of the piezoelectric sensor junction (connecting the head to the holder) for the first and subsequent modes were performed in the study, resonance conditions were determined, and their impact on the sensor’s operation was assessed. The aim of the calculations was to optimize the material and shape of the junction so that it does not resonate with modes that would interfere with the sensor’s operation while being of simple and technologically feasible design.

Vibrational modeling of the junction was conducted, and the results of the modeling matched the calculations within the limits of negligible error.

A prototype of a piezo sensor was constructed and experimentally tested. This type of piezo sensor was found to be suitable for testing ultrasonic systems. A specific prototype constructed for experiments successfully tested ultrasonic systems vibrating at a frequency of 20 kHz–96 kHz. The tested systems were not lighter than 55 g, and the optimal pressure force of the sensor needle was 0.8–1.2 N.

## Figures and Tables

**Table 1:** Results of calculating resonant frequencies of junction vibrations.

Mode (Hz)	Nature of Junction end Fixation			
	Both Ends Are Supported	Both Ends Are Fixed	Both Ends Are Free	One End Is Free, and the Other Is Supported
Main frequency	522.9	1184.5	1194.8	818.6
Second	2091.9	3264.9	3264.9	2646.2
Third	4706.8	6401.4	6401.4	5518.7
Fourth	8367.6	10,580.9	10,580.9	9449.2
Fifth	13,074.4	15,805.0	15,805.0	14,412.9

## Data Availability

The data that support the findings of this research are available from the author upon reasonable request.
